# miR-497 and miR-34a retard lung cancer growth by co-inhibiting cyclin E1 (CCNE1)

**DOI:** 10.18632/oncotarget.3693

**Published:** 2015-03-29

**Authors:** Zhiyuan Han, Yanbin Zhang, Qiaoyuan Yang, Binbin Liu, Jianjun Wu, Yajie Zhang, Chengfeng Yang, Yiguo Jiang

**Affiliations:** ^1^ State Key Laboratory of Respiratory Disease, Institute for Chemical Carcinogenesis, Guangzhou Medical University, Guangzhou, PR China; ^2^ Department of Pulmonary Tuberculosis, Guangzhou Chest Hospital, Guangzhou, PR China; ^3^ Department of Pathology, Guangzhou Medical University, Guangzhou, PR China; ^4^ Department of Physiology and Center for Integrative Toxicology, Michigan State University, East Lansing, MI, USA

**Keywords:** CCNE1, lung cancer, miR-34a, miR-497

## Abstract

Cyclin E1, encoded by the CCNE1 gene, promotes G1/S transition, chromosome instability, and oncogenesis. Here, we show that miR-497 and miR-34a target the 3′-UTR of *CCNE1*. miR-497 and miR-34a are downregulated in cancer cells and their ectopic expression inhibited cell proliferation and colony formation in vitro, and inhibited tumor growth in a xenograft model. The effect of simultaneous overexpression of miR-497 and miR-34a on the inhibition of cell proliferation, colony formation, and tumor growth, and the downregulation of cyclin E1 was stronger than the effect of each miRNA alone. The synergistic actions of miR-497 and miR-34a partly correlated with cyclin E1 levels. When cells stably expressing *CCNE1* were transfected with the Hi-miR-497/34a plasmid, there was no effect on colony formation, compared with that of cells transfected with either Hi-miR497 or Hi-miR34a. These results indicate cyclin E1 is downregulated by both miR-497 and miR-34a, which synergistically retard the growth of human lung cancer cells.

## INTRODUCTION

Cancer is a complex disease caused by the progressive accumulation of genetic and epigenetic alterations in cells, which allow the cells to evade normal and environmental controls. Much progress has been made in the treatment of lung cancer in the last 10 years. Nonetheless, lung cancer is currently the most common cause of cancer-related death throughout the world, and therefore remains an unresolved medical issue. An understanding of the processes and pathogenesis of cancer at the systemic, cellular, and molecular levels is one of the most ambitious goals of cancer research. MicroRNAs (miRNAs) are epigenetic regulators that play a pivotal role in the acquisition of tumorigenic properties by cells.

miRNAs are a class of endogenous noncoding RNAs of approximately 22 nucleotides (nt) that regulate mRNA stability and translation [1-3]. A wide range of biological functions are controlled by miRNAs, including cell proliferation, differentiation, and apoptosis [4-6]. There is strong evidence that miRNAs can act as oncogenes or tumor suppressors, with key roles in cancer initiation, progression, and therapy. In an attempt to understand the mechanisms underlying cancer, an increasing number of studies have reported that individual miRNAs exert their functions in specific cancers. Many recent studies reported that some miRNAs cooperatively control a variety of biological processes, including cell development and differentiation, apoptosis, and the cell cycle [7-10]. When an mRNA or several different mRNAs involved in a specific biological process are targeted by several miRNAs, the miRNAs act cooperatively. Recent studies have demonstrated that miRNAs encoded in miRNA clusters function synergistically in cancer, e.g., the miR-17-92 cluster [11-13] and the miR-15a-16-1 cluster [14, 15]. Ventura et al. reported that miR-17-92 and miR-106b-25 double-knockout mice have a more severe phenotype than miR-17-92 single-knockout mice [16].

In our previous study [17], we profiled miRNA expression in lung cancer cells treated with resveratrol, a potential cancer chemopreventive agent. We found nine miRNAs (miR-622, -512-5p, -504, -497, -34a, -302d, -302b*, -29c, -20b) upregulated in the cells. Only miR-622, -497, and -34a inhibited the proliferation of lung cancer cells, whereas the remaining six miRNAs had no effect on cell proliferation in our preliminary experiments. miR-497 and -34a have a similar seed sequence, indicating that they might share similar functions; therefore, in the present study, these miRNAs were selected for the identification of potential targets and to explore their roles in lung cancer. Bioinformatics analysis suggested that both miRNAs target the *CCNE1* gene, which encodes Cyclin E1. Cyclin E1, a member of the conserved cyclin E family, activates CDK2 [18] and regulates the transition of mammalian cells from quiescence to S phase. Transgenic cyclin E1 triggers dysplasia and multiple pulmonary adenocarcinomas [19, 20], and the overexpression of cyclin E1 was suggested to contribute to cancer development or tumorigenesis in various types of cancer, including breast, colon, and lung cancers [21]. Increased expression of cyclin E1 is a useful marker of poor prognosis in lung cancer [22]. These data suggest that cyclin E1 is a potential target for the treatment of lung cancer. Based on previous data, we designed a study to test the hypothesis that cyclin E1 expression is coregulated by miR-497 and miR-34a in lung cancer.

## RESULTS

### miR-497 and miR-34a inhibit the proliferation of human lung cancer cells

**Figure 1:**
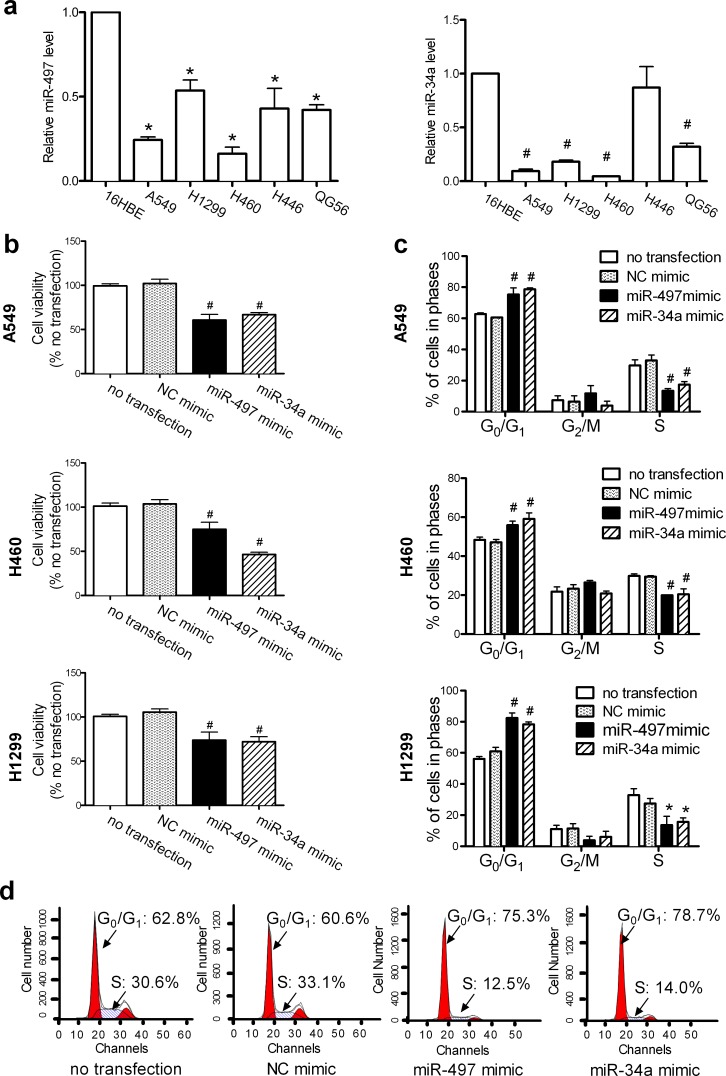
Elevated levels of miR-497 or miR-34a inhibit cell proliferation (**a**) The relative levels of miR-497 and miR-34a were determined with the TaqMan^®^ MicroRNA Assay and are expressed as fold change after normalization to the internal control, U6BsnRNA. Mean ± SEM, n = 3 (*P < 0.05, #P < 0.01, all vs. 16HBE cells). (**b**) A549, H460, and H1299 cells were transfected as described in Methods, and cell growth was monitored at 48 h using the CCK-8 assay. Mean ± SD, n = 3 (#P < 0.01 vs. NC mimic). (**c**) A549, H460, and H1299 cells were transfected, harvested at 48 h, and stained for cell cycle analysis on a FACSCalibur flow cytometer. Mean ± SD, n = 3 (*P < 0.05, #P < 0.01, all vs. NC mimic). (**d**) Representative histograms show the elevated levels of miR-497 and miR-34a that induced G0/G1 cell-cycle arrest in A549 cells; the percentages of cells in G0/G1 phase and S phase are shown.

miR-34a is downregulated in lung cancer tissues and cells [23, 24]; however, few reports have examined the expression of miR-497 in lung cancer. Although miR-497 is downregulated in lung cancer [25, 26], its specific role remains to be determined. Analysis of the expression of miR-497 and miR-34a in lung cancer cells showed that the levels of miR-497 and miR-34a (Figure 1a) were reduced by 24.29 ± 2.50% and 9.43 ± 2.96% in A549, 16.11 ± 5.20% and 4.51 ± 0.34% in H460, 53.55 ± 9.28% and 18.25 ± 2.14% in H1299, 43.00 ± 15.46% and 87.01 ± 27.73% in H446, and 42.17 ± 4.26% and 32.04 ± 4.58% in QG56 lung cancer cells, respectively, compared to those in normal bronchial epithelial 16HBE cells.

The cell viability of A549, H460, and H1299 lung cancer cells was decreased by 66.71 ± 1.65%, 46.36 ± 1.96% and 72.10 ± 4.02 %, respectively, in response to miR-34a overexpression, and by 60.71 ± 4.63%, 74.94 ± 3.58%, and 73.71 ± 6.50%, respectively, in response to miR-497 overexpression (Figure 1b). Downregulation of the expression of miR-34a (Figure S1a) or miR-497 (Figure S1b) with inhibitors had no effect on the growth of A549, H460, and H1299 cells (Figure S1c) because the endogenous levels of these miRNAs in these cells are low. To identify the phase of the cell cycle at which the miRNAs exert their proliferation-inhibitory effect, cell-cycle distribution was analyzed by flow cytometry. Transfection with miR-497 or miR-34a mimics caused cell-cycle arrest at G_0_/G_1_ phase in A549, H1299, and H460 lung cancer cells (Figure 1c). Typical histograms of the cell-cycle arrest induced by miR-497 or miR-34a in A549 cells are shown in Figure 1d.

### miR-497 and miR-34a suppress colony formation and tumorigenesis

**Figure 2:**
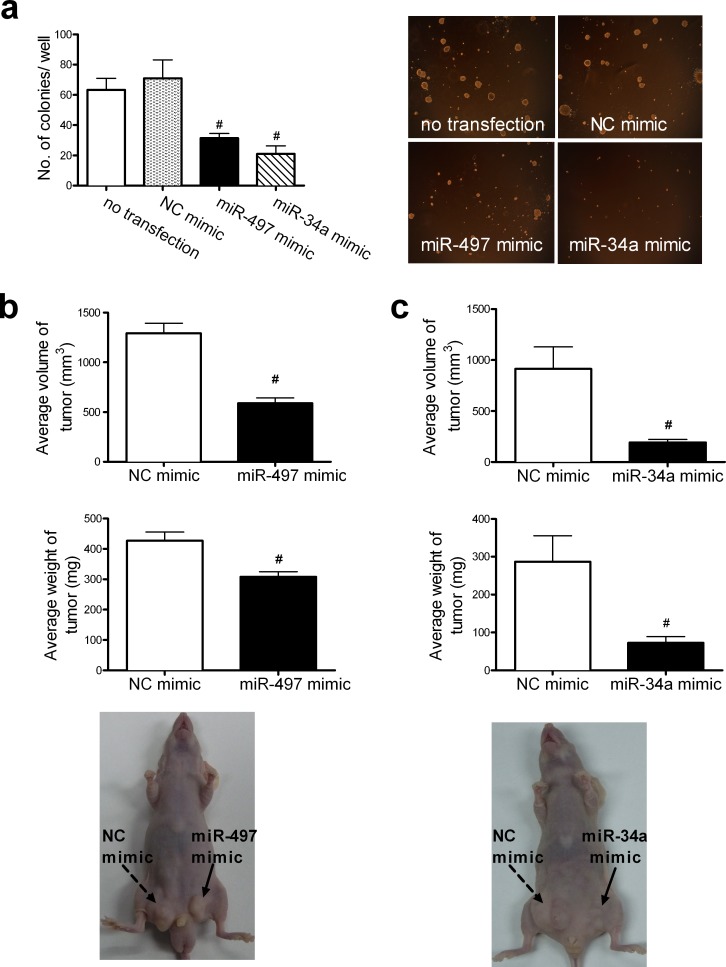
Elevated levels of miR-497 and miR-34a retard cell growth *in vitro* and *in vivo* (**a**) Colony formation by A549 cells transfected with the miR-497 mimic or the miR-34a mimic was examined in soft agar. The number of colonies per well (≥50 cells per colony) in triplicate wells is shown in the left column. Mean ± SD (#P < 0.01 vs. NC mimic). A representative result of the colony-formation assay is shown in the right column (original magnification, ×40). The effects of miR-497 (**b**) or miR-34a (**c**) on tumor formation were examined in a nude mouse xenograft model. The bilateral inguinoabdominal flanks of nude mice were injected s.c. with NC-mimic-transfected A549 cells (left flank) and miR-497-mimic-transfected A549 cells (right flank), or with NC-mimic transfected A549 cells (left flank) and miR-34a-mimic-transfected A549 cells (right flank). The miR-497-mimic-transfected or miR-34a-mimic-transfected cells generated tumors with smaller volumes and lower weights, as determined at necropsy, than those of tumors generated with NC-mimic-transfected cells in the contralateral flanks. Mean ± SD, n = 7 (#P < 0.01, all vs. NC mimic). Photographs illustrating the features of tumor growth at necropsy are shown at the bottom of Fig. 2b and 3c.

The effect of miR-497 and miR-34a on the colony forming ability of A549 cells was assessed. Cells transfected with miR-497 or miR-34a mimics showed fewer (31.33 ± 2.44 and 21.00 ± 4.00 colonies per well, respectively) and smaller colonies than those observed in the control groups (71.00 ± 9.33 colonies per well) (Figure 2a).

The effect of miR-497 or miR-34a on tumorigenicity was examined in vivo. The bilateral inguino-abdominal flanks of nude mice were inoculated subcutaneously (s.c.) with A549 cells transfected with normal control (NC) (left flank) or miR-497 (right flank) mimics or with NC (left flank) or miR-34a (right flank) mimics. At 5 weeks, the average volumes (588.39 ± 117.34 mm^3^) and weights (308.57 ± 26.53 mg) of tumors in groups of seven mice injected with cells transfected with miR-497 mimics were lower than those (1293.16 ± 198.57 mm^3^ and 427.14 ± 365.31 mg) in the control group injected with the NC mimic (Figure 2b), while the average volumes (190.25 ± 67.79 mm^3^) and weights (72.86 ± 31.84 mg) of tumors in mice injected with cells transfected with miR-34a mimics were lower than those (913.14 ± 455.23 mm^3^ and 287.20 ± 131.09 mg) in the control group injected with the NC mimic (Figure 2c). This evidence collectively suggests that miR-497 and miR-34a inhibit cell growth and might function as tumor suppressors.

### CCNE1 is a putative target of miR-497 and miR-34a

Three bioinformatics algorithms (Targetscan 5.0, RNAhybrid 2.1, and RNA22) predicted that miR-497 and miR-34a target *CCNE1*, which encodes the cyclin E1 protein. The 535-nt 3′ untranslated region (UTR) of *CCNE1* was screened for complementarity to the seed sequences of miR-497 and miR-34a. Two predicted target sequences for miR-497 were identified at nt 223–254 and nt 467–492 (Figure S2a). The putative secondary RNA hybrids, with minimum free energy (ΔG), are shown in Figure S2b. All ΔG values were approximately −20.0 kcal/mol, which is considered authentic for miRNA targets. The miR-497 target sequences at nt 223–254 and nt 467–492 of the *CCNE1* 3-UTR are highly conserved among nine species (Figure S2c). One predicted target sequence for miR-34a was found at nt 226–255 (Figure S2d). Figure S2e shows the putative secondary RNA hybrid, with its ΔG. The miR-34a target sequence at nt 226–255 of the 3′-UTR is highly conserved among nine species (Figure S2f). Although the sequence of the miR-34a seed region pairs with G:U complementarity at nt 247, 248, and 253 of the UTR, the seed regions of miR-497 (5′-AGCAGCA-3′) and miR-34a (5′-GGCAGUG-3′) are complementary to the same sequence at nt 247–253 (5′-UGCUGCU-3′) in the UTR. Therefore, miR-497 and miR-34a share one binding site (nt 247–253) in the 3′-UTR of *CCNE1*.

### CCNE1 is a direct target of miR-497 and miR-34a

**Figure 3:**
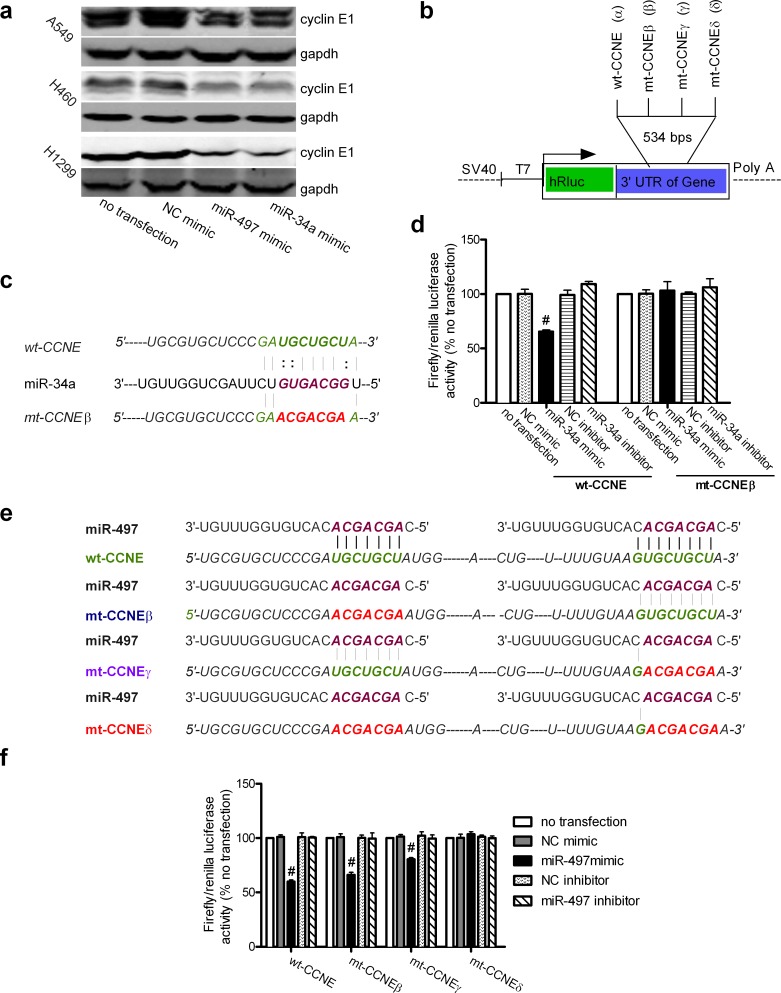
CCNE1 is a direct target of miR-497 and miR-34a (**a**) The expression levels of the cyclin E1 protein were measured by immunoblotting using GAPDH as the loading control. Experiments were performed in triplicate, with results similar to those shown. (**b**) Diagram of the psiCHECK2 vector used for the luciferase reporter activity assay. The arrow indicates the transcription start site. The magnified panel shows the location of the wild-type CCNE1 3′-UTR (α, wt-CCNE) or mutant CCNE1 3′-UTR at nt 247–253 (β, mt-CCNEβ), at nt 483–490 (γ, mt-CCNEγ), and at both (δ, mt-CCNEδ), which were subcloned into the psiCHECK2 vector. Thus, four individual plasmids were generated: wt-CCNE, mt-CCNEβ, mt-CCNEγ, and mt-CCNEδ. (**c**) Diagram of the wt-CCNE and mt-CCNEβ reporter constructs. mt-CCNEβ contains a seven-base mutation in the miR- 34a target region, abolishing its binding to miR-34a. (**d**) Firefly luciferase reporter activity assay. The relative luciferase activity was normalized to the Renilla luciferase activity and compared with that in the no-transfection control. Mean ± SD, n = 3 (#P < 0.01 vs. NC mimic). (**e**) Outline of the wt-CCNE, mt-CCNEβ, mt-CCNEγ, and mt-CCNEδ reporter constructs. mt-CCNEβ and mt-CCNEγ each contain one seven-base mutation in the miR-497 target region (at nt 247–253 or nt 483–490, respectively, in the CCNE1 3′-UTR), partly abolishing their binding to miR-497. mt-CCNEδ contains two seven-base mutations in the miR-497 target regions (at nt 247–253 and nt 483–490 of the CCNE1 3′-UTR), completely abolishing its binding to miR-497. (**f**) Firefly luciferase reporter activity assay. Relative luciferase activity was normalized to the Renilla luciferase activity and compared with that in the no-transfection control. Mean ± SD, n =3 (#P < 0.01, all vs. NC mimic).

To confirm that *CCNE1* is targeted by miR-497 and miR-34a, we investigated the effects of miR-497 and miR-34a on cyclin E1 levels by immunoblotting. Overexpression of miR-497 or miR-34a in A549, H460, and H1299 lung cancer cells by transfection with miR-497 or miR-34a mimics (Figure S3a and S3b) markedly reduced the levels of cyclin E1 protein (Figure 3a). However, real-time quantitative polymerase chain reaction (real-time qPCR) showed no changes in *CCNE1* mRNA levels in response to miR-497 or miR-34a upregulation (Figure S3c). This indicates that the negative correlation between cyclin E1 expression and miR-497 or miR-34a levels is due to post-transcriptional modulation of cyclin E1 expression.

The wild-type *CCNE1* 3′-UTR and a mutant *CCNE1* 3′-UTR were subcloned downstream from the *Renilla* luciferase gene in the psiCHECK2 vector to determine whether they bound directly to seed regions of the miRNAs. In Figure 3b, the wild-type sequence (α, wt-CCNE), mutant sequences at nt 247–253 (β, mt-CCNEβ) or nt 485–491 (γ, mt-CCNEγ), and mutant sequences at both binding sites (δ, mt-CCNEδ) in the *CCNE1* 3′-UTR were inserted individually into the vector, generating four plasmids as follows: wt-CCNE, mt-CCNEβ, mt-CCNEγ, and mt-CCNEδ. The binding sites between miR-34a and the wild-type or mutant *CCNE1* were examined (Figure 3c). When the wt-CCNE plasmid was cotransfected with the miR-34a mimic, the luciferase activity of A549 cells was markedly reduced by 34.56 ± 1.13% (Figure 3d). When the mt-CCNEβ plasmid was cotransfected with the miR-34a mimic or an inhibitor, the luciferase activity did not differ from that of the control. These data confirm that miR-34a directly targets *CCNE1*. To examine the interaction between miR-497 and *CCNE1*, the binding sites in *CCNE1* were completely mutated, or mutated individually at each nucleotide (Figure 3e). When the wt-CCNE plasmid was cotransfected with the miR-497 mimic, the luciferase activity was markedly reduced by 40.09 ± 1.99% (Figure 3f). Moreover, when either plasmid (mt-CCNEβ or mt-CCNEγ) with only one mutant site was cotransfected with the miR-497 mimic, the luciferase activity was slightly reduced, whereas cotransfection of the mt-CCNEδ plasmid, which contained two completely mutated binding sites, with the miR-497 mimic reversed the miR-497 induced reduction of luciferase activity (Figure 3f). However, because the endogenous levels of miR-34a and miR-497 are low in A549 cells, transfection with miR-497 and miR-34a inhibitors did not affect the levels of luciferase activity (Figures 3d and 1f).

### Cyclin E1 mediates the effects of miR-497 or miR-34a on cell growth

**Figure 4:**
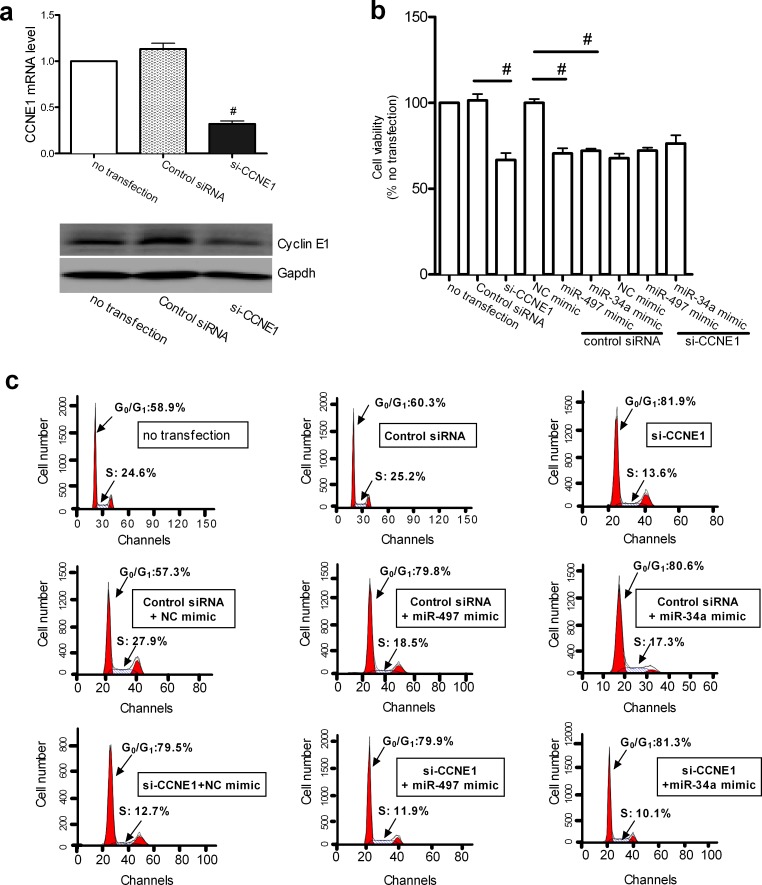
CCNE1 knockdown affected the miR-497 and miR-34a induced inhibition of proliferation (**a**) Validation of siRNA-mediated CCNE1 silencing in A549 cells. The relative CCNE1 mRNA levels, as determined by real-time qPCR, are expressed as fold changes after normalization to the internal control (18S rRNA) (upper row). Mean ± SEM, n = 3 (#P < 0.01 vs. control siRNA). GAPDH was used as the loading control for immunoblotting to determine the cyclin E1 protein levels. A representative result from three independent experiments is shown (bottom row). (**b**) Cell growth was monitored in A549 cells using the CCK-8 assay. Mean ± SD, n = 3 (#P < 0.01, CCNE1 siRNA vs. control siRNA, control siRNA + miR-497 mimic vs. control siRNA + NC mimic, control siRNA + miR-34a mimic vs. control siRNA + NC mimic). (**c**) Cell-cycle distribution of G0/G1 phase in A549 cells was analyzed with a FACSCalibur flow cytometer. Representative histograms show the percentages of cells in the G0/G1 and S phases.

Because *CCNE1* was identified as a direct target of miR-497 and miR-34a, we investigated whether the effects of miR-497 and miR-34a on cell proliferation were mediated by the modulation of *CCNE1* expression. Cell proliferation and cell-cycle distribution were examined in A549 cells after *CCNE1* knockdown and overexpression of the miRNAs. The efficiency of RNA interference was confirmed by real-time qPCR (Figure 4a, upper panel) and immunoblotting (Figure 4a, lower panel). Knockdown of *CCNE1* significantly inhibited cell viability and G_0_/G_1_ arrest in A549 cells, whereas miR-497 or miR-34a overexpression did not enhance the effect of *CCNE1* knockdown (Figure 4b and 4c). A typical histogram of the cell cycle is shown in Figure 4c. These results support the assumption that the effects of miR-497 and miR-34a on cell growth are mediated by its modulation of *CCNE1* expression.

### Synergistic effects of miR-497 and miR-34a on tumor growth retardation

**Figure 5:**
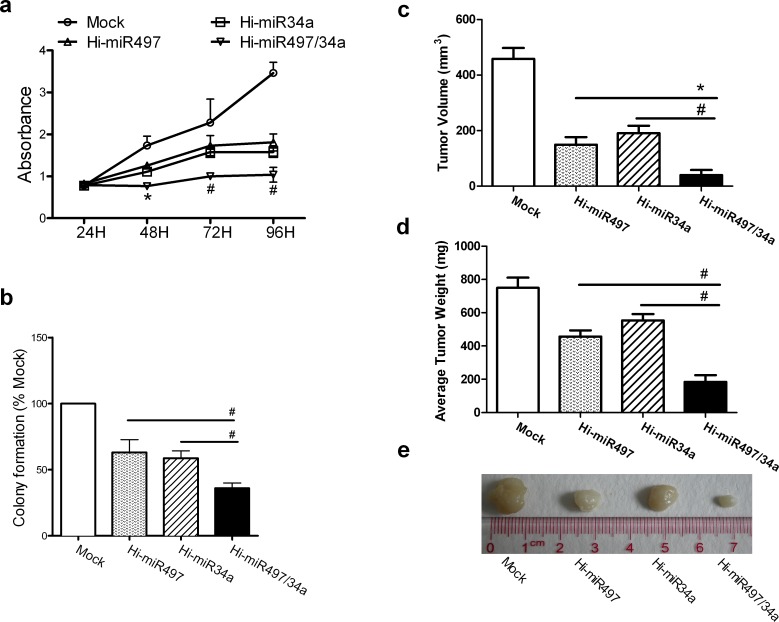
miR-497 and miR-34a synergistically retard cell growth (**a**) The cell-growth curves for A549 cells transfected with Hi-miR497, Hi-miR-34a, or Hi-miR497/34a at 24, 48, 72, and 96 h. Means ± SD, n = 3 (*P < 0.05, at 48 h, Hi-miR497/34a vs. Hi-miR497;#P < 0.01, at 72 and 96 h, Hi-miR497/34a vs. Hi-miR497 or Hi-miR34a). (**b**) A549 cells were transfected with Hi-miR497, Hi-miR34a, or Hi-miR497/34a. Colony formation was examined in soft agar. Numbers of colonies per well (≥50 cells per colony) in triplicate wells are shown. Mean ± SD (#P < 0.01, Hi-miR497/34a vs. Hi-miR497, Hi-miR497/34a vs. Hi-miR34a). The cooperative effects of miR-497 and miR-34a on tumor formation were examined in a nude mouse xenograft model. Hi-miR497/34a-transfected A549 cells were injected s.c. into the right inguino-abdominal flanks of nude mice. The Hi-miR497/34a-transfected cell treatment generated tumors with smaller volumes (**c**) and lower tumor weights (**d**), as determined at necropsy, than those of tumors generated with mock-transfected cells. Mean ± SD, n = 5 (#P < 0.01, Hi-miR497/34a vs. Hi-miR497, Hi-miR497/34a vs. Hi-miR34a). (**e**) Images show the features of tumor growth at necropsy.

Because *CCNE1* is a cotarget of miR-497 and miR-34a, we examined whether the two miRNAs exert synergistic effects on cell growth. First, expression plasmids transiently expressing miR-497 (Hi-miR497), miR-34a (Hi-miR-34a), and a plasmid coexpressing miR-497 and miR-34a (Hi-miR497/34a) were constructed and verified by DNA sequencing and a TaqMan^®^ MicroRNA Assay (Figure S4). The viability of cells transiently transfected with Hi-miR497, Hi-miR-34a, or Hi-miR497/34a was reduced at 48, 72, and 96 h. At 72 h, cell viability decreased from 2.28 ± 0.17 in the mock group to 1.73 ± 0.17, 1.58 ± 0.09, or 1.0 ± 0.02 in cells transfected with Hi-miR497, Hi-miR-34a, or Hi-miR497/34a, respectively, indicating that the Hi-miR497/34a plasmid caused a more marked reduction in cell viability than the Hi-miR497 or Hi-miR-34a plasmids (Figure 5a). The proliferation inhibition rate of Hi-miR497/34a (55.99%) was almost the same as the total inhibition rate (54.82%) of Hi-miR497 and Hi-miR-34a at 72 h. The colony formation rate of cells transiently transfected with Hi-miR497, Hi-miR-34a, or Hi-miR497/34a decreased by 36.84 ± 7.02%, 41.23 ± 4.09%, or 64.04 ± 2.92% (Figure 5b). Cells transfected with miR-497/miR-34a formed fewer and smaller colonies than cells transfected with Hi-miR-34a or Hi-miR497 alone. A549 cells transiently transfected with mock (empty plasmid), Hi-miR497, Hi-miR34a, or Hi-miR497/34a were inoculated s.c. into the bilateral inguino-abdominal flanks of nude mice. The average volume of tumors expressing Hi-miR497 (149.40 ± 17.84 mm^3^), Hi-miR-34a (190.80 ± 19.36 mm^3^), or Hi-miR497/34a (39.60 ± 14.32 mm^3^) was lower than that of the mock group (458.20 ± 30.64 mm^3^) (Figure 5c). The average weight of tumors expressing Hi-miR497 (456.00 ± 27.20 mg), Hi-miR-34a (554.00 ± 28.80 mg), or Hi-miR497/34a (184.00 ± 28.80) was lower than that of the mock group (750.00 ± 40.00 mg) after 5 weeks (Figure 5d). This indicated that the average volume and average weight of tumors transfected with Hi-miR497/34a were lower than those in the other groups after 5 weeks. Figure 5e illustrates the features of tumor growth. The tumor weight inhibition rate of Hi-miR497/34a (75.47%) was greater than the total inhibition rate (65.33%) induced by Hi-miR497 and Hi-miR-34a, whereas the tumor volume inhibition rate of Hi-miR497/34a was comparable to the total inhibition rate of Hi-miR497 and Hi-miR-34a. Taken together, these data indicate that miR-497 and miR-34a cooperate in inhibiting tumor growth.

### Synergistic effects of miR-497 and miR-34a on cotargeting *CCNE1*

**Figure 6:**
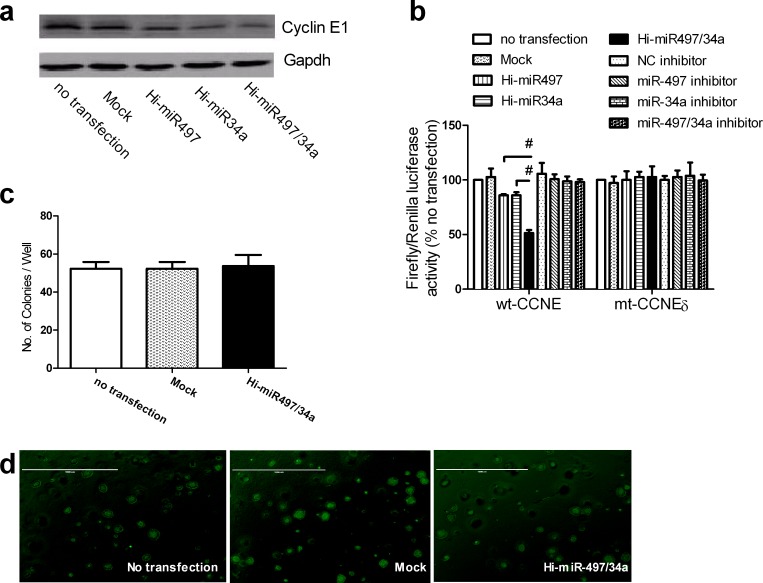
miR-497 and miR-34a act synergistically by cotargeting CCNE1 (**a**) Cyclin E1 protein levels in individually transfected A549 cells. Cyclin E1 protein levels were determined by immunoblotting using GAPDH as the loading control. A representative result of three independent experiments is shown. (**b**) Firefly luciferase reporter activity assay. A549 cells were transfected with Hi-miR497, Hi-miR34a, Hi-miR497/34a, or inhibitors. The relative luciferase activity was normalized to the Renilla luciferase activity and compared with that of the untransfected control. Mean ± SD, n = 3 (#P < 0.01 Hi-miR497/34a vs. Hi-miR497 or Hi-miR34a). (**c**) Hi-CCNE1a cells were transfected with empty vector or Hi-miR497/34a. Colony formation was examined in soft agar. (**d**) A representative result of the colony formation assay is shown (original magnification, ×40).

A549 cells stably expressing cyclin E1 (designated Hi-CCNE1a) were generated (Figure S5). Hi-CCNE1-a cells transfected with Hi-miR497/34a expressed lower levels of cyclin E1 protein than those transfected with Hi-miR497 or Hi-miR34a (Figure 6a). This suggests that the coexpression of miR-497 and miR-34a enhanced the effect of each individual miRNA on the modulation of cyclin E1 expression. A reporter gene activity assay was used to test this hypothesis in A549 cells. Cotransfection of the wt-CCNE plasmid with Hi-miR497/34a reduced luciferase activity to a greater extent than cotransfection of wt-CCNE with Hi-miR497 or Hi-miR34a alone (Figure 6b). However, when the mt-CCNEδ plasmid was cotransfected with Hi-miR497/34a, the luciferase activity did not differ from that of cells cotransfected with either Hi-miR497 or Hi-miR34a alone (Figure 6b). This suggests that the synergistic effects of miR-497 and miR-34a are correlated with the levels of cyclin E1. Transfection of Hi-CCNE1a cells with Hi-miR-497/34a did not affect colony formation (Figure 6c) compared with that of cells transfected with either Hi-miR497 or Hi-miR34a, as shown in Figure 6d. Therefore, overexpression of *CCNE1* abolished the growth retardation induced by miR497 and miR34a in A549 cells, and *CCNE1* mediates the synergistic effects of miR-497 and miR-34a.

## DISCUSSION

The major finding of our study is that miR-497 and miR-34a synergistically inhibit the expression of the same gene, *CCNE1*, and the function of its encoded protein, cyclin E1, thereby impeding the growth of lung cancer cells. Several studies have demonstrated that miR-34a retards lung cancer cell growth or induces apoptosis by targeting TGFβR2 [23], Axl [27], Notch-1 [28], or HDM4 [29], whereas miR-497 does so by targeting HDGF [26] in lung cancer. Our study extends the results of others by showing that miR-34a and miR-497 cotarget *CCNE1* in lung cancer cells. To the best of our knowledge, this is the first study to demonstrate the cooperative effect of miR-497 and miR-34a, which belong to different miRNA families, on inhibiting cancer cell growth.

In our previous study [17], we reported that an antitumor agent, resveratrol, upregulates the expression of several miRNAs (including miR-497 and miR-34a), and thus might inhibit the proliferation of lung cancer cells. This prompted us to investigate whether these two miRNAs function as antitumor agents. Some miRNAs that are downregulated in cancer are found in regions with frequent loss of heterozygosity [30]. miRNA-497 and miRNA-34a are located on chromosomes 17p13.1 and 1p36, respectively. The loss or deletion of chromosome 17p13.1 or 1p36 has been reported in various types of cancer, including lung cancer [31, 32], suggesting that the downregulation of miRNA-497 or miRNA-34a in these cancers arises from genomic DNA loss or deletion. miR-34a, located at 1p36, has been extensively studied as a microtumor suppressor [33, 34], and miR-497, located at 17p13.1, is deleted in 93% of small-cell lung cancers [35]. Son et al. reported that miR-497 is downregulated in lung cancer [25]; however, its role in lung cancer remains unclear. Here, we studied the function of miR-497 in lung cancer cells, and showed that miR-497 and miR-34a inhibit cell growth in vitro and in vivo, supporting our hypothesis that the two miRNAs function as tumor suppressors. Several studies have reported that miR-497 and miR-34a are potential anticancer agents based on their ability to target oncogenes [24, 25, 36, 37]. In the present study, we found that they cotarget *CCNE1*, repressing the expression of cyclin E1.

Cyclin E1 is an essential regulator of cell cycle G_1_ progression and entry into S phase. It is a nuclear protein that was first identified by its ability to complement the proliferative defects of cyclin-deficient yeast cells. It is overexpressed in various cancers, including lung, breast, and gastrointestinal tract tumors [38-40]. Several mechanisms regulate its expression in tumors. Although gene amplification might be involved, the expression of cyclin E1 is also modulated post-transcriptionally by miRNAs. Cyclin E1 (or cyclin E) is repressed by miR-15b, miR-16, miR-34c, and miR-145 [41, 42] in other cancer cells. In the present study, we showed for the first time that both miR-34a and miR-497 bind to the 3′-UTR region of *CCNE1*, as confirmed by immunoblotting and luciferase activity assays. Previous studies showed that miR-34a targets the oncogenes *CDK4*, *CDK6*, *CCND1*, *MET*, and *BCL2* [36, 43, 44], whereas miR-497 targets the oncogenes *CCND2* and *BCL2* [37, 45] in various types of cancer. These and our results, together with the strong possibility that more miR-497 or miR-34a targets will be discovered soon, suggest that these two miRNAs regulate proliferation-related mRNAs (including *CCNE1*), and thus function as tumor suppressors.

The protein encoded by *CCNE1*, a newly identified cotarget for miR-34a and miR-497, plays a key role in regulating the growth of lung cancer cells, and its effect is mediated by the cooperative action of the two miRNAs. Several studies have focused on the effects of the miRNA family axis on cancer because many miRNAs contain the same 7-nt or 8-nt seed sequence. Most single miRNA mutations do not markedly affect the corresponding phenotype, whereas knocking out multiple or all miRNA family members can affect several phenotypes in insects [46, 47]. This phenomenon, which reflects the complexity of miRNAs and especially the miRNA–miRNA network, allows them to perform many biological functions. Here, we found that two miRNAs from different families synergistically inhibit the growth of lung cancer cells, which indicates that the two miRNA families share similar biological functions. Biochemical and bioinformatics studies showed that the 5′ ends of miRNAs, designated as “seed sequences”, play critical roles in target recognition and posttranscriptional repression [3, 48, 49]. The recognition is based on the complementary base paring between the seed sequence and the target mRNA. Studies have shown that G:U base pairing is tolerated in seed complementarity [3, 50]. Therefore, miR-497 and miR-34a, with seed sequences 5′-AGCAGCA-3′ and 5′-GGCAGUG-3′, respectively, can bind to the same sequence (3′-UCGUCGU-5′) in the 3′ UTR of *CCNE1*; however, there are three-base variations between their seed regions. This was verified in our luciferase activity assay. Our results indicated that miR-497 and miR-34a synergistically inhibit cell proliferation, predominantly by repressing the expression of their cotarget, *CCNE1*. However, the relationship between miR-497 and miR-34a is complex and requires further research.

Activation of cyclin E:Cdk2 results in retinoblastoma tumor-suppressor gene (RB) inactivation by hyper-phosphorylation, induction of E2F target gene transcription, and cell cycle G1/S transition [18]. Cell cycle dysregulation (including the overexpression of cyclins) occurs frequently in neoplasia or cancer. Multiple reports suggested that cyclin E is a promising therapeutic target in lung [20, 51], ovarian [52], and breast cancers [53, 54]. Li et al [53] showed that cyclin E siRNA delivered by intratumoral injection effectively inhibits cyclin E expression in vivo and results in tumor suppression. However, there are limitations to the therapeutic application of siRNAs, such as their stability, off-target side effects, and interferon responses to foreign nucleic acids. Unlike siRNAs targeting a single molecule, the miRNA approach might have a greater potential as cancer therapeutics because of the endogenous nature of miRNAs and their capacity to simultaneously regulate many different oncogenes across multiple pathways. Furthermore, evidence indicates that a combination of two tumor-suppressive miRNAs is superior to single miRNAs for the repression of oncogene expression, the inhibition of proliferation and invasion of cancer cells in culture, and the inhibition of tumor proliferation in vivo. Our data indicated that the tumor-suppressive miR-34a and miR-497 could co-inhibit cyclin E1, suggesting that cyclin E1 is a key target mediating the anti-tumor effect of miR-34a and miR-497 in lung cancer. Both miRNAs modulate other oncogenic genes that may mediate their tumor suppressor function. Chek1 (putative target of miR-497 identified by the algorithm DIANA miRPath v.2.0), cdc25a (miR-497) [55], and cdk6 (miR-497and miR-34a) [55, 56] are involved in the indirect or direct regulation of the cyclin E1 downstream genes cdk2, RB and E2f3 (target for miR-34a, [33, 57]). Therefore, the combination of miR-34a and miR-497 could be superior to each individual miRNA in its ability to retard lung cancer cell growth to some extent.

In conclusion, we showed that miR-497 and miR-34a act cooperatively to regulate certain aspects of tumorigenesis, including the growth of lung cancer cell lines, especially through their cooperative effect on the downregulation of cyclin E1 expression.

## MATERIALS AND METHODS

### Cell culture and reagents

The lung cancer cell lines A549, H1299, H460, H446, and QG56, purchased from the Chinese Academy of Sciences Cell Bank of Type Culture Collection (Shanghai, China), were cultured in RPMI-1640 medium (Invitrogen, Carlsbad, CA) supplemented with 10% fetal bovine serum (FBS; Sijiqing Co., Ltd, Hangzhou, China). Normal human bronchial epithelial cells (16HBE), kindly provided by Dr. Xujun from the Guangzhou Institute of Respiratory Diseases, were maintained in minimum essential medium supplemented with 10% FBS (Sijiqing Co., Ltd). All cells were incubated at 37°C in a humidified atmosphere of 5% CO_2_. Antibody directed against cyclin E1 (Santa Cruz Biotechnology, Santa Cruz, CA), Alexa-Fluor-680-conjugated goat anti-mouse IgG antibody (Molecular Probes, Invitrogen), and IRDye 800-conjugated anti-rabbit IgG antibody (Li-Cor, Lincoln, NE) were purchased for immunoblotting.

### Transient transfection of miRNAs or small interfering RNA (siRNA) oligonucleotides

The oligonucleotides including miRNA mimics, inhibitors, and their negative control oligos were purchased from GenePharma (Shanghai, China). Cells were transiently transfected with 50 nM miR-497 mimic, miR-34a mimic, miR-497 inhibitor, miR-34a inhibitor, CCNE1 siRNA (Qiagen, Germany), or siRNA negative control (NC siRNA; Qiagen) using Lipofectamine 2000 (Invitrogen).

### RNA extraction and real-time qPCR

Total RNA was extracted with the TRIzol Reagent (Invitrogen). miRNA expression was analyzed using the TaqMan® MicroRNA Assay (Ambion, Austin, TX), which detects mature miRNAs, on the ABI 7500 Real-Time PCR System (Applied Biosystems, Foster City, CA), according to the manufacturer's protocol. Real-time qPCR was used to confirm the expression levels of mRNAs. cDNA was produced according to the protocol for PrimeScript™ RT Reagent (TaKaRa, Japan), and real-time qPCR was performed as described in the method for SYBR® Premix Ex Taq™ II (TaKaRa) with the Rotor-Gene 6000™ (Corbett Research), supplied with analytical software. 18S rRNA was used for normalization. The oligonucleotides used as PCR primers were as follows: CCNE1 (forward) 5′-CGTGGCCTCTAAGATGAAGG-3′, CCNE1 (reverse) 5′-CTGGCATTTTGGAGAGGAAG-3′; 18S rRNA (forward) 5′-TCAGTGGTGGACCTGACCTG-3′, 18S rRNA (reverse) 5′-TGCTGTAGCCAAATTCGTTG-3′.

### Immunoblotting analysis

Total cell lysates were prepared with cell lysis buffer (CST, Beverly, MA) containing proteinase inhibitors (1% cocktail and 1 mmol/L PMSF, both from Kangcheng, China). Cell proteins (50 μg) were separated by 12% SDS–PAGE and transferred to PVDF membranes (Millipore, Bedford, MA). The membranes were blocked, incubated with anti-cyclin E1 or anti-glyceraldehyde phosphate dehydrogenase (GAPDH) antibody, and then with secondary antibodies. Immunoblotting analyses were performed with the Odyssey Infrared Imaging System (Li-Cor).

### Luciferase assays

The sequences of the *CCNE1* 3′-UTR and the *CCNE1* 3-UTRs in which the putative binding sites had been mutated were amplified with specific primers (listed in Table S1), and verified by DNA sequencing. These gene fragments were then subcloned downstream from the *Renilla* luciferase gene in the psiCHECK2 vector (Promega, Madison, WI) to generate the wild-type *CCNE1* plasmid (wt-CCNE), the partial mutant *CCNE1* plasmids (mt-CCNEβ and mt-CCNEγ), and the complete mutant *CCNE1* plasmid (mt-CCNEδ). For luciferase assays, A549 cells were transfected with wt-CCNE, mt-CCNEβ, mt-CCNEγ, or mt-CCNEδ in 24-well plates using Lipofectamine 2000 (Invitrogen). The transfection mixtures contained 100 ng of plasmid and 50 nM synthetic mimic, inhibitor, Hi-miR497, Hi-miR34a, or Hi-miR497/34a. A549 cells were also transfected with the psiCHECK-2 vector as the normalization control. The cells were collected 48 h after transfection, and the luciferase activity was measured with the Dual-Luciferase Reporter Assay System (Promega).

### Cell viability and cell-cycle assays

For the cell viability assay, cells were incubated in 10% Cell Counting Kit-8 (CCK-8; Dojindo, Japan), diluted in normal culture medium, at 37°C for 2 h and the absorbance at 490 nm/absorbance at 650 nm (A_490/650_) ratio was calculated. For cell-cycle analysis, cells were washed twice with PBS, collected, fixed, stained with propidium iodide (Sigma, St Louis, MO), and analyzed with fluorescence-activated cell sorting (FACS) on a FACSCalibur flow cytometer (Becton Dickinson, Mountain View, CA). Cell viability and cell-cycle distribution were determined 48 h after transfection.

### Soft-agar colony formation

Transfected cells were propagated on soft agar. A base layer (1.5 mL) of agar (Amresco, Solon, OH) (0.6% agar in RPMI-1640 with 10% FBS) was allowed to solidify in a six-well flat-bottomed plate before the addition of 2 mL of a cell suspension containing 2000 cells in 0.3% agar in RPMI-1640 with 10% FBS. The colonies were allowed to grow for 28 days at 37°C under 5% CO_2_ and visualized with an inverted microscope (Olympus IX71, Olympus, Tokyo, Japan).

### Tumor xenograft model

Five-week-old male nude mice (BALB/c nu/nu), purchased from the Medical Animal Experimental Center of Guangdong Province, were used to examine the tumorigenicity of the transfected cells. The animal protocol was approved by the Animal Care and Use Committee. The transfected cells were propagated and 5 × 10^6^ cells were inoculated s.c. into the dorsal flanks of the mice. Tumor size was measured weekly and tumor volume was estimated as described in our previous report [17]. Tumors were removed and weighed 5 weeks after the injection of tumor cells.

### Expression vector construction

The sequences of the miR-497 precursor (MIR497) and the miR-34a precursor (MIR34A) were amplified with oligonucleotide primers synthesized by Invitrogen (Figure S4). MIR497 and MIR34A were then inserted individually into the pcDNA™6.2-GW/EmGFPmiR vector (Invitrogen) using the BLOCK-iT™ Pol II miR RNAi Expression Vector Kit (Invitrogen), to generate the expression constructs Hi-miR497 and Hi-miR34a, respectively. These were verified by DNA sequencing with the primer 5′- CTCTAGATCAACCACTTTGT-3′ in an ABI Prism 373 Genetic Analyzer (Applied Biosystems). The empty pcDNA™6.2-GW/EmGFPmiR vector was used as the negative control (mock). To investigate the cooperative activities of miR-497 and miR-34a, we used the isocaudomers *Bam*HI (NEB, Beverly, MA) and *Bgl*II (NEB) to ligate both MIR497 and MIR34A into the vector to generate the expression construct Hi-miR497/34a, which was confirmed by DNA sequencing.

A clone of the green fluorescent protein (GFP)-tagged cDNA [including complete 3-UTR encoding human *CCNE1* (NM_001238.2)], was bought from Origene (Rockville, MD) as transfection-ready plasmid DNA. The neomycin-resistance gene is expressed downstream from the SV40 promoter in the same vector, which permits the positive selection of transfected cells. To construct cells stably expressing *CCNE1*, A549 cells were transfected with the plasmid DNA and selected with neomycin (0.5 mg/mL).

### Statistical analysis

All assays were performed in triplicate in three independent experiments, and all data are expressed as the mean ± SD. Statistical differences between two groups were evaluated with the two-tailed Student's *t* test and analysis of variance (ANOVA) was used for multiple comparisons. In all cases, *P* < 0.05 was considered significant. All statistical tests were performed with the statistical analysis software IBM SPSS Statistics 20 for Mac (International Business Machines Corporation, New York, NY).

## SUPPLEMENTARY MATERIALS, FIGURES, TABLES



## Abbreviations

miRNA: microRNA
3′-UTR: 3′-untranslated region
FBS: fetal bovine serum
siRNA: small interfering RNA
CCK-8: cell counting kit-8
NC inhibitor: miRNA inhibitor negative control
NC mimic: miRNA mimic negative control
qRT-PCR: quantitative real-time polymerase chain reaction
RT: reverse transcription
SD: standard deviation
