# Supplementary material for: miR-497 and miR-34a retard lung cancer growth by co-inhibiting cyclin E1 (CCNE1)
Source: Oncotarget. 2015 Mar 29;6(15):13149–63. doi: 10.18632/oncotarget.3693 (PMC4537005; doi:10.18632/oncotarget.3693)
Supplement: Supplementary file 1 [file oncotarget-06-13149-s001.pdf]

miR-497 and miR-34a retard lung cancer growth by co-inhibiting cyclin E1 (CCNE1)

Supplementary Information

Supplementary Table S1. Oligo primers for CCNE1 3'UTR and its mutant sequence

3'UTR	Oligo primers	Template	Sequence of primers	Sequence of amplified products
wt-CCNE	wt-CCNE-F	cDNA from A549	5'CCG ctcgagCCACCCCATCCTTCTCCACCAAAG 3'	CCACCCCATCCTTCTCCACCAAAGACAGTTGCGCGCCTGCTCCACGTTCTCTTCTGTCTGTTGCAGCGGAGGCGTG<br>CGTTTGCTTTTACAGATATCTGAATGGAAGAGTGTTTCTTCCACAACAGAAGTATTTCTGTGGATGGCATCAAACAGGG<br>CAAAGTGTTTTTTATTGAATGCTTATAGGTTTTTTTTAAATAAGTGGGTCAAGTACACCAGCCACCTCCAGACACCAGTG<br>CGTGCTCCCGA**TGCTGCT**ATGGAAGGTGCTACTTGACCTAAGGGACTCCCACAACAACAAAAGCTTGAAGCTGTGGA<br>GGGCCACGGTGGCGTGGCTCTCCTCGCAGGTGTTCTGGGCTCCGTTGTACCAAGTGGAGCAGGTGGTTGCGGGCA<br>AGCGTTGTGCAGAGCCCATAGCCAGCTGGGCAGGGGGCTGACCTCTCCACATTATCAGTTGACAGTGTACAATGCCT<br>TTGATGAACTGTTTTGTAAG**TGCTGCT**ATATCTATCCATTTTTTAATAAAGATAATACTGTTTTTGAGACA
	wt-CCNE-R		5'ATAAGAAT gcggccgcTGTCTCAAAAACAGTATTATCTTTATTAAAAAATGGA 3'	
mt-CCNEβ	mt-CCNEβ-F	wt-CCNE vector	5'CACCAGTGCGTGCTCCCGAACGACGAATGGAAGGTGCTACTTGAC 3'	CCACCCCATCCTTCTCCACCAAAGACAGTTGCGCGCCTGCTCCACGTTCTCTTCTGTCTGTTGCAGCGGAGGCGTG<br>CGTTTGCTTTTACAGATATCTGAATGGAAGAGTGTTTCTTCCACAACAGAAGTATTTCTGTGGATGGCATCAAACAGGG<br>CAAAGTGTTTTTTATTGAATGCTTATAGGTTTTTTTTAAATAAGTGGGTCAAGTACACCAGCCACCTCCAGACACCAGTG<br>CGTGCTCCCGA**ACGACGA**ATGGAAGGTGCTTACTTGACCTAAGGGACTCCCACAACAACAAAAGCTTGAAGCTGTG<br>GAGGGCCACGGTGGCGTGGCTCTCCTCGCAGGTGTTCTGGGCTCCGTTGTACCAAGTGGAGCAGGTGGTTGCGGG<br>CAAGCGTTGTGCAGAGCCCATAGCCAGCTGGGCAGGGGGCTGACCTCTCCACATTATCAGTTGACAGTGTACAATGC<br>CTTTGATGAACTGTTTTGTAAG**TGCTGCT**ATATCTATCCATTTTTTAATAAAGATAATACTGTTTTTGAGACAGC
	mt-CCNEβ-R		5'GTCAAGTAGCACCTTCCATTCGTCGTTCGGGAGCACGCACTGGTG 3'	
mt-CCNEγ	mt-CCNEγ-F	wt-CCNE vector	5'GATGAACTGTTTTGTAAGACGACGAATATCTATCCATTTTTTAATA 3'	CCACCCCATCCTTCTCCACCAAAGACAGTTGCGCGCCTGCTCCACGTTCTCTTCTGTCTGTTGCAGCGGAGGCGTG<br>CGTTTGCTTTTACAGATATCTGAATGGAAGAGTGTTTCTTCCACAACAGAAGTATTTCTGTGGATGGCATCAAACAGGG<br>CAAAGTGTTTTTTATTGAATGCTTATAGGTTTTTTTTAAATAAGTGGGTCAAGTACACCAGCCACCTCCAGACACCAGTG<br>CGTGCTCCCGA**TGCTGCT**ATGGAAGGTGCTACTTGACCTAAGGGACTCCCACAACAACAAAAGCTTGAAGCTGTGGA<br>GGGCCACGGTGGCGTGGCTCTCCTCGCAGGTGTTCTGGGCTCCGTTGTACCAAGTGGAGCAGGTGGTTGCGGGCA<br>AGCGTTGTGCAGAGCCCATAGCCAGCTGGGCAGGGGGCTGCCCTCTCCACATTATCAGTTGACAGTGTACAATGCCT<br>TTGATGAACTGTTTTGTAAG**ACGACGA**ATATCTATCCATTTTTTAATAAAGATAATACTGTTTTTGAGACAGC
	mt-CCNEγ-R		5'TATTAAAAAATGGATAGATATTCGTCGTCTTACAAAACAGTTCATC 3'	
mt-CCNEδ	mt-CCNEδ-F	mt-CCNEβ vector	5'GATGAACTGTTTTGTAAGACGACGAATATCTATCCATTTTTTAATA 3'	CCACCCCATCCTTCTCCACCAAAGACAGTTGCGCGCCTGCTCCACGTTCTCTTCTGTCTGTTGCAGCGGAGGCGTG<br>CGTTTGCTTTTACAGATATCTGAATGGAAGAGTGTTTCTTCCACAACAGAAGTATTTCTGTGGATGGCATCAAACAGGG<br>CAAAGTGTTTTTTATTGAATGCTTATAGGTTTTTTTTAAATAAGTGGGTCAAGTACACCAGCCACCTCCAGACACCAGTG<br>CGTGCTCCCGA**ACGACGA**ATGGAAGGTGCTACTTGACCTAAGGGACTCCCACAACAACAAAAGCTTGAAGCTGTGG<br>AGGGCCACGGTGGCGTGGCTCTCCTCGCAGGTGTTCTGGGCTCCGTTGTACCAAGTGGAGCAGGTGGTTGCGGGC<br>AAGCGTTGTGCAGAGCCCATAGCCAGCTGGGCAGGGGGCTGCCCTCTCCACATTATCAGTTGACAGTGTACAATGCC<br>TTTGATGAACTGTTTTGTAAG**ACGACGA**ATATCTATCCATTTTTTAATAAAGATAATACTGTTTTTGAGACAGC
	mt-CCNEδ-R		5'TATTAAAAAATGGATAGATATTCGTCGTCTTACAAAACAGTTCATC 3'	

Note：1，F and R in Oligo primers column stand for forward and reverse primers; 2, Blue and red in the last column stand for wild type- and mutant type-sequence of binding sites

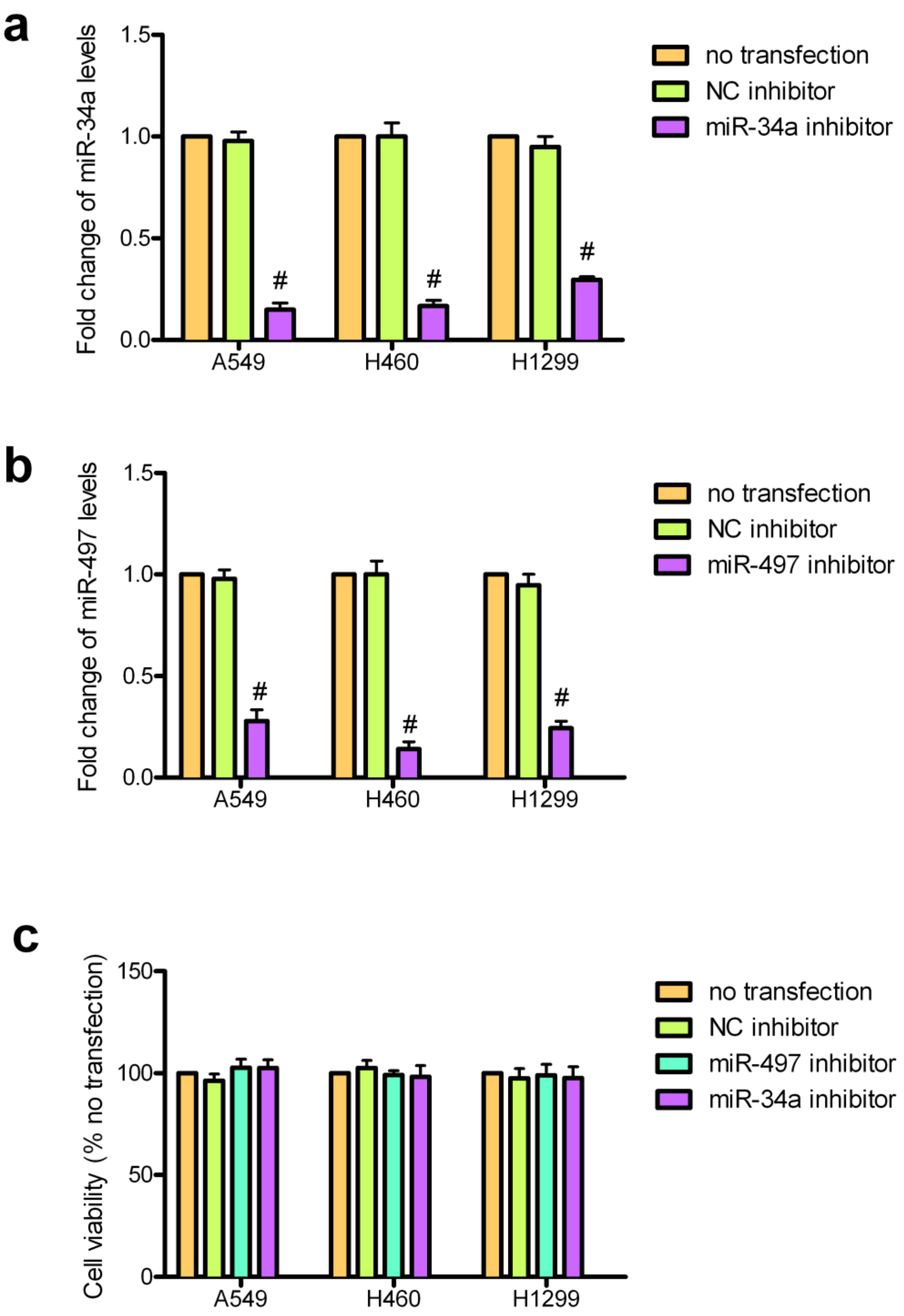

Figure S1: Effect of miR-497 or miR-34a knockdown on cell proliferation. The relative miR-497 (a) or miR-34a (b) levels in A549, H460, and H1299 cells were assessed with the TaqMan® MicroRNA Assay and are expressed as fold change after normalization to the internal control, U6B-snRNA. Mean ± SD, n = 3 (#P < 0.01, all

vs. NC inhibitor). (c) A549, H460, and H1299 cells were transfected as described in the Methods section, and cell growth was monitored for 48 h using the CCK-8 assay. There were no differences among the groups. Mean ± SD, n = 3.

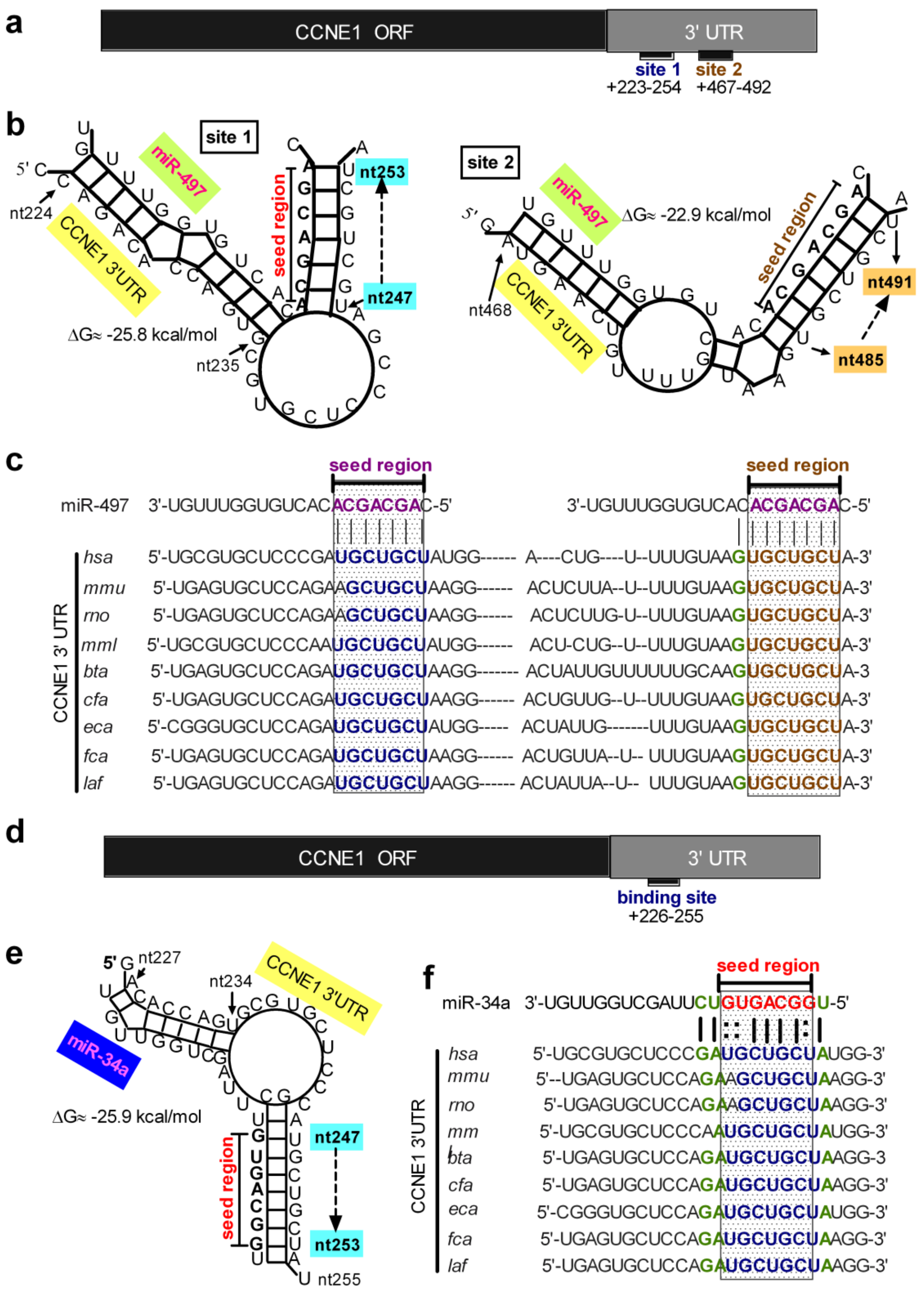

Figure S2: Bioinformatics analysis of miR-497 and miR-34a target sequences in the CCNE1 3′-UTR. (a) The locations of the two putative miR-497 target sites are shown. (b) Two putative binding sites and miR-497:CCNE1 RNA hybrids, as predicted from their minimum free energy (ΔG), are illustrated. (c) Comparison of the miR-497 seed sequence and its target sequences in nine species. (d) The location of the putative miR-34a target site is shown. (e) The putative binding site and miR-34a:CCNE1 RNAhybrid, as predicted from the ΔG, are illustrated. (f) Comparison of the miR-34a seed sequence and its target sequences in nine species.

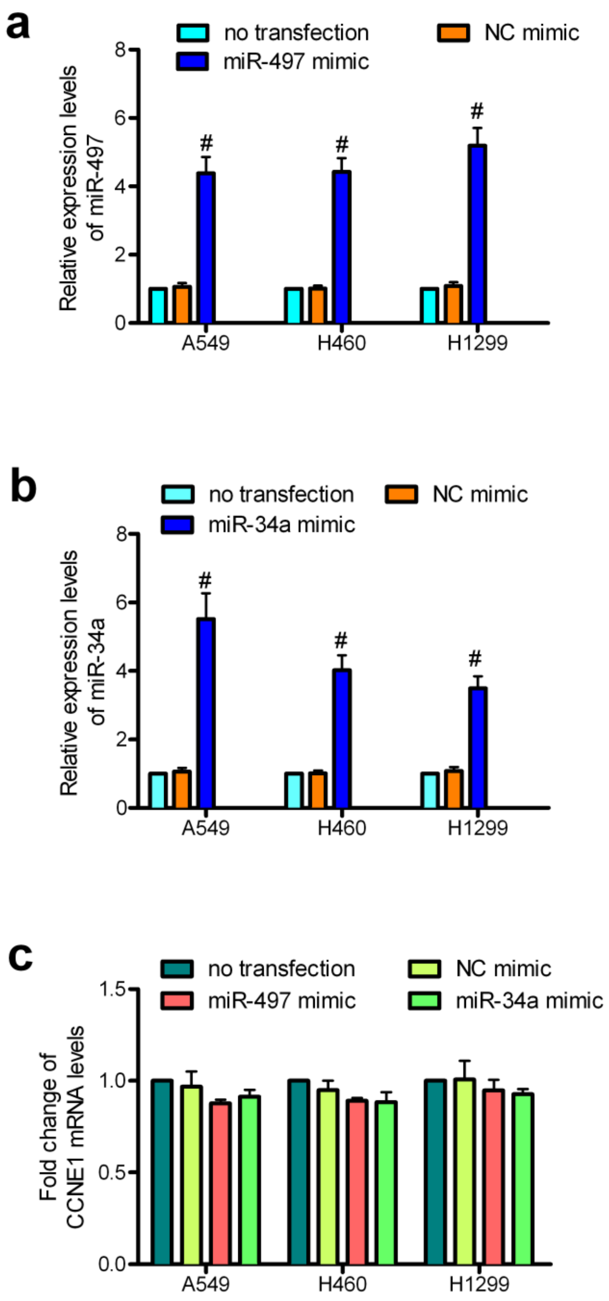

Figure S3: Effect of miR-497 or miR-34a overexpression on CCNE1 mRNA levels. The relative miR-34a (a) or miR-497 (b) levels in A549, H460, and H1299 cells were assessed with the TaqMan® MicroRNA Assay and are expressed as fold change after normalization to the internal control, U6B-snRNA. Mean ± SD, n = 3 (#P < 0.01, all vs. NC mimic). (c) The relative CCNE1 mRNA levels in A549, H460, and H1299 cells were assessed by real-time qPCR and are expressed as fold change after normalization to the internal control, 18S rRNA. Mean ± SD, n = 3.

a

Oligo primers	Sequences
MIR497F	TGCTGcagcagcacacTgTggTTTgTGTTTTGGCCACTGACTGACAcAAAccAgtgtgctgctg
MIR497R	CCTGcagcagcacacTggTTTgTGTCAGTCAGTGGCCAAAACAcAAAccAcAgtgtgctgctgC
MIR34AF	TGCTGTggcagTgTcTTagcTggTTgTGTTTTGGCCACTGACTGACAcAAccAgAAgAcActgccA
MIR34AR	CCTGTggcagTgTcTTcTggTTgTGTCAGTCAGTGGCCAAAACAcAAccAgctAAgAcActgccAC

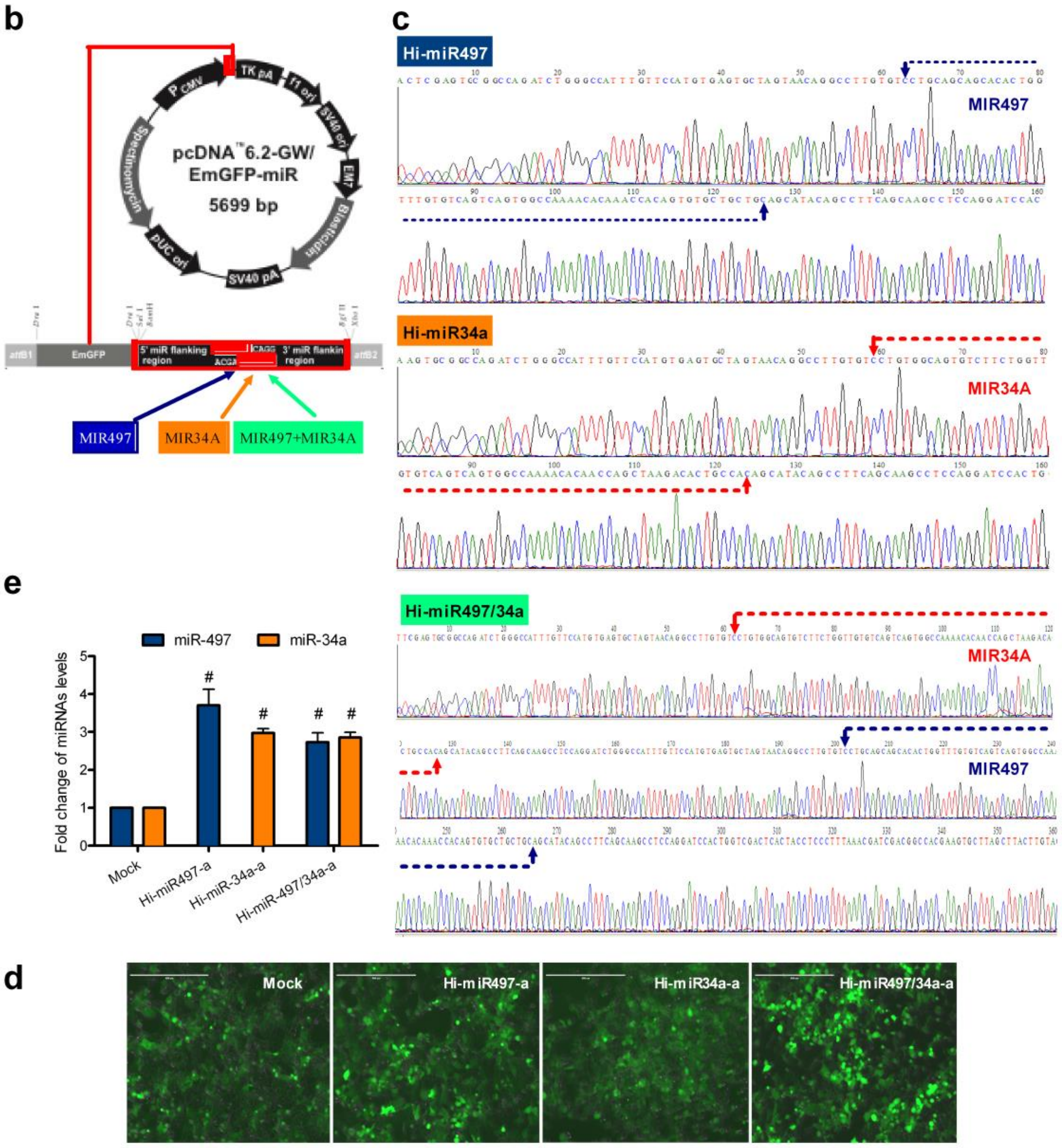

Figure S4: miRNA expression plasmid construction. (a) Oligonucleotide primers for the synthesis of miR-497 and miR-34a precursors (MIR497 and MIR34A, respectively). (b) Diagram of the pcDNA6.2-GW/EmGFP-miR vector used to

construct the expression plasmids. The red region indicates the transcription start site. The magnified panel shows the locations of MIR497, MIR34A, or both precursors, which were subcloned into the pcDNA6.2-GW/EmGFP-miR vector. The isocaudomers BamHI and BglII were used to ligate MIR497 and MIR34A into the vector. (c) The partial sequences of the Hi-miR497 (top, in dark blue), Hi-miR34a (middle, in red), and Hi-miR497/34a (bottom row) vectors, which were confirmed by DNA sequencing, are shown. (d) A549 cells were transfected with the expression plasmid Hi-miR497, Hi-miR34a, or Hi-miR497/34a, generating HimiR497-a, Hi-miR34a-a, or Hi-miR497/34a-a cells, respectively. The empty pcDNA6.2-GW/EmGFP-miR vector was used as the negative control (mock), (original magnification, ×40). (e) The relative miR-497 and miR-34a levels were determined with the TaqMan® MicroRNA Assay and are expressed as fold change after normalization to the internal control, U6B-snRNA. Mean ± SD, n = 3 (#$P < 0.01$, all vs. mock).

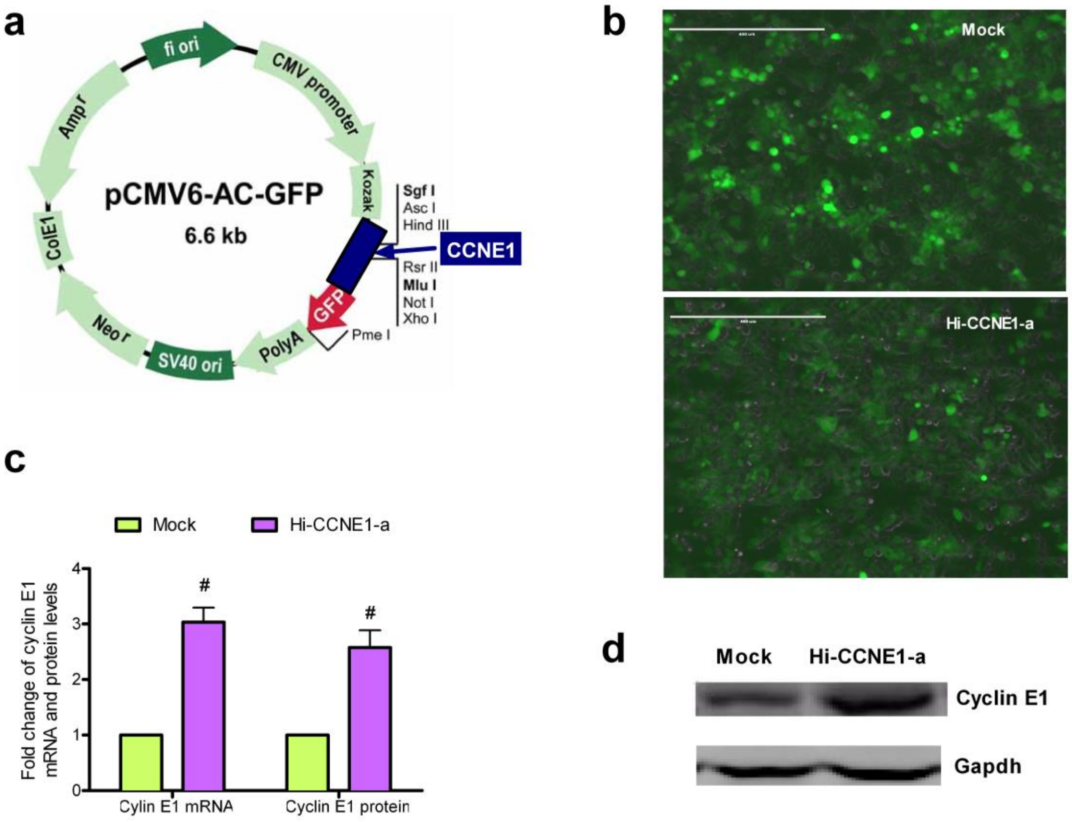

Figure S5: To construct cells stably expressing CCNE1, A549 cells were transfected with plasmid DNA and the positive clones stably expressing CCNE1 (Hi-CCNE1a) were selected with neomycin. (a) A clone of the GFP-tagged cDNA (including the UTR) encoding human CCNE1, which was purchased as transfection-ready plasmid DNA from Origene, is illustrated. (b) Representative images of the Hi-CCNE1a cells were captured with an Olympus BX51 microscope with an attached CCD camera (original magnification, ×40). Validation of the stable expression of CCNE1 in Hi-CCNE1a cells. (c) The relative CCNE1 mRNA levels were measured by real-time qPCR and are expressed as fold change after normalization to the internal control, 18S rRNA (top row). Mean ± SEM, n = 3 (#P < 0.01 vs. control siRNA). GAPDH was used as the loading control for immunoblotting to determine cyclin E1 protein levels. (d) A result representative of three independent experiments is shown.
